# Experimental GVD engineering in slow light slot photonic crystal waveguides

**DOI:** 10.1038/srep26956

**Published:** 2016-05-31

**Authors:** Samuel Serna, Pierre Colman, Weiwei Zhang, Xavier Le Roux, Charles Caer, Laurent Vivien, Eric Cassan

**Affiliations:** 1 Institut d’Electronique Fondamentale, Univ Paris-Sud, CNRS UMR 8622, Université Paris Saclay, Bat. 220, 91405 Orsay Cedex, France; 2 Laboratoire Charles Fabry, Institut d’Optique Graduate School, CNRS, Université Paris Saclay, 2 Avenue Augustin Fresnel, 91127 Palaiseau Cedex, France; 3 Zurich Research Laboratory, IBM Research GmbH, Ruschlikon 8803, Switzerland

## Abstract

The use in silicon photonics of the new optical materials developed in soft matter science (e.g. polymers, liquids) is delicate because their low refractive index weakens the confinement of light and prevents an efficient control of the dispersion properties through the geometry. We experimentally demonstrate that such materials can be incorporated in 700 μm long slot photonic crystal waveguides, and hence can benefit from both slow-light field enhancement effect and slot-induced ultra-small effective areas. Additionally, we show that their dispersion can be engineered from anomalous to normal regions, along with the presence of multiple zero group velocity dispersion (ZGVD) points exhibiting Normalized Delay Bandwidth Product as high as 0.156. The reported results provide experimental evidence for an accurate control of the dispersion properties of fillable periodical slotted structures in silicon photonics, which is of direct interest for on-chip all-optical data treatment using nonlinear optical effects in hybrid-on-silicon technologies.

The maturity of the Silicon on Insulator (SOI) platform allows the fabrication of submicrometer cross-section photonic waveguiding structures with low propagation losses while benefiting from a natural compatibility with the CMOS technology^1^. Besides, nonlinear phenomena in photonic integrated circuits have been considered as a promising route to ensure important functionalities like modulation or commutation at higher rates than their electronic counterparts, especially by using third-order nonlinear optical effects in silicon^2^,^3^. In order to realize small footprint optical components with low energy consumption for the all-optical processing of information, it is required to enhance the light matter interactions in small volumes. In this purpose, photonic crystal waveguides (PhCWs) appear as good candidates due to their confinement and versatile dispersion properties including slow light and possible control of group velocity dispersion (GVD) effects^4^,^5^,^6^. Additionally, there is in these slow light structures a further enhancement of the nonlinearity that depends on the group index (*n*_*G*_). This behaviour is quantified by the slow down factor enhancement defined as S = *n*_*G*_/*n*, with *n* the material refractive index^7^. Third order nonlinear effects such as Kerr self-phase modulation- scale with *S*^2^, hence proportionally to the square of the group index^8^,^9^. Consequently, slow light structures are of great interest due to their improvement of the nonlinear device performance.

However, the bandwidth of these arrangements becomes narrower as the group index (<*n*_*G*_>) increases, and without engineering of the waveguide the large GVD distorts the optical wavefront^10^ and severely limits the efficiency of nonlinear effects. Moreover, light propagation loss increases in the slow light regime due to back and out-of-plane scattering^11^. For example, considering a W1 plain waveguide, where the fabrication errors are minor compared to its slotted counterpart, a dramatic increase of extrinsic optical losses is observed for large group indices. It has indeed been shown that even for waveguides as short as 50–100 μm, the system departs from perfect Bloch-modes^12^ at ng ≈ 50, hence capping the maximal usable slow light. A simple figure of merit to estimate the intrinsic performances of a slow light structure, namely its buffering capacity and its suitability for nonlinear operation such as broad-band Four-wave mixing, is the Normalized Delay Bandwidth Product (NDBP)^9^, defined as NDBP ≈ <*n*_*G*_> Δω/ω, where <*n*_*G*_> is calculated within 10% variation with respect to the mean group index value^13^,^14^. Finally, even in the range of moderate propagation losses for *n*_*G*_ < 40, this optimistic picture of enhanced optical nonlinearities by slow light phenomena is severely counterbalanced in silicon by free carriers. At telecommunication wavelengths, strong two photon absorption (TPA) in silicon and its associated effects related to the presence of a free carrier plasma indeed spoil the Kerr effect^3^. This last limitation for nonlinear applications is intrinsic to the use of silicon-based photonic crystals^15^, as explained by models considering both multiphoton absorption and linear scattering effects^16^.

To circumvent this problem, other materials with better nonlinear figures of merit than silicon, such as GaInP, have been explored^17^. Nevertheless the costs of integrability make solutions based of silicon still of prior interest. In this prospect, hybrid silicon waveguides based on a hollow core slot geometry have been proposed and demonstrated, first in a classical picture^18^ and later within a PCW scheme^19^. In the versatile toolbox of PhCWs, these slotted (SPhCWs) configurations have brought the possibility to add up slow light and GVD-nearly-on-demand properties of PhCWs with hollow core waveguide geometries.

At the present time, the dispersion properties of SPhCWs have been numerically explored with interesting results^20^,^21^, mainly in free-standing configurations. Yet, they remain essentially unexplored experimentally. The reasons for this stem firstly from the sensitivity of SPhCWs dispersion properties on the fabricated geometrical parameters. For instance, some of the theoretical proposals rely in exotic shapes or asymmetries that are difficult to control during the fabrication processes^21^,^22^. Secondly, it is difficult to probe experimentally the slow light and dispersive features of the waveguides. Though, the control of slow-light and GVD in SPhCWs is essential in order to fully benefit from the hybrid integration of soft matter (and low index) materials on silicon (e.g. polymers, nanocomposites). Furthermore, compared to their standard plain counter-parts, hybrid slotted structures are of greater interest due to their even smaller effective areas^23^.

In this context, this paper reports experimental results demonstrating the control of the group index, GVD sign and bandwidth in long SPhCW (700 μm) through a proper control of the waveguide geometrical parameters. These results are in agreement with numerical 3D-Plane Wave Expansion calculations. We stress that all experiments are carried out in SPhCWs on SOI (i.e. non-free-standing air membranes) where the holes^24^ and the slot have been filled with an index liquid in order to mimic the integration of soft matter materials such as polymers or doped compounds^25^,^26^ in hybrid waveguides in the purpose of relying on a mechanically robust and viable integration PhC-on-silicon scheme.

We designed SPhCWs for given dispersion properties not only by the control of their hole positions but also by accounting for the slot width and the filling material index. Experimental evidence of dispersion engineered is provided. Namely, quite large bandwidths are reported in the slow light regime (NDBP up to 0.156). An Optical Coherent Tomography (OCT) based method is used^27^ in order to directly measure the optical delay and to estimate the group index as a function of wavelength without the need of any fitting parameter. As a whole, the experimental results open the concrete possibility to control the GVD properties in order to enhance third order nonlinear optical effects through slow-light boost, while preserving the phase matching condition (quasi-flat band) for broadband four wave mixing^28^.

## Results

### Design and fabrication

The device geometry consists in a 260 nm silicon thin film SOI wafer with lattice constant *a* = 420 nm, and hole radius *r* = 125 nm (0.30*a*) to ensure a wide TE bandgap. In order to pre-tune the dispersion curve, the first and second rows of holes are shifted towards and outwards the slot by 0.20*a* and 0.35*a*, respectively^29^. As the optical mode is mainly contained between these two rows of holes, their geometry and position directly impact the dispersion properties. Additionally, the position of the holes is easier to control during fabrication than their size and shape. Under these premises, the radius of the second row of holes is kept fixed to 125 nm and the radius of the first row (*r*_1_) is swept between 95 nm (0.23a) and 125 nm (0.3a) in order to quantify the experimental effects deriving from the modification of only one parameter. The structures are fabricated through electronic beam lithography (details are given in the methods section). After a mechanical cleaving, a Cargille liquid with refractive index of 1.45 at 1550 nm is drop casted on top of the sample.

Frequency band engineering of the proposed structure is studied first numerically. The physical explanations of the related phenomena are then given by comparing the electric-field distributions of several modified PhC waveguides.

**Figure 1:**
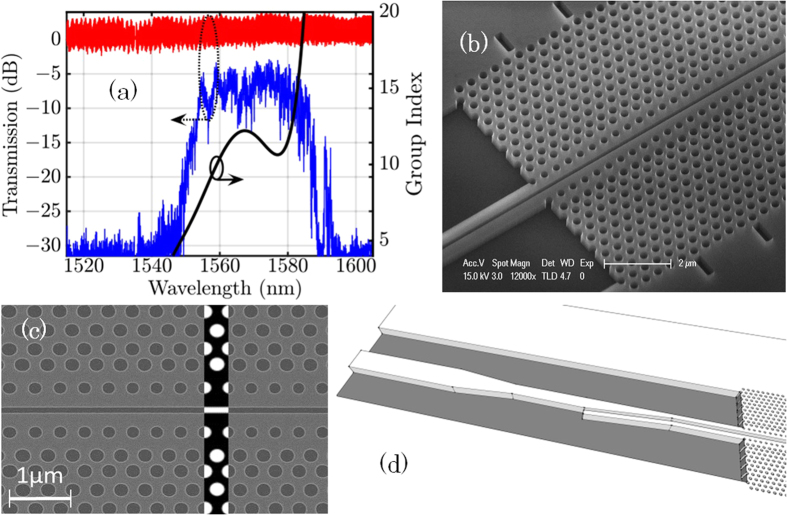
(**a**) Linear transmission of the 700 μm SPhCW, including coupling loss between the access waveguide and the SPhCW. (**b**) SEM image of the SPhCW with coupling tapers. (**c**) SEM view of the fabricated structure. Numerical design is surimposed in black and white. (**d**) View of the coupling taper beween the access plain waveguide and the slot PhCW.

Figure 1 shows the transmission of a 700 μm-long SPhCW and, as an element of comparison, the transmission of the access strip waveguide which has typical propagation loss lower than 1 dB/cm. Light injection into the slow light slotted waveguide mode requires a specific design of injection stages in order to make the mode profile and group index mismatches decrease^30^, both being responsible for high insertion losses. Starting from the sample edge (butt-coupled scheme), the design includes a wide strip access waveguide whose size is gradually minimized into a narrow single-mode strip waveguide, followed by a strip-to-slot converter^31^. Finally, we used at the input of the photonic crystal two transition regions of five periods each (440 nm and 430 nm lattice long) in order to adapt the group index mismatch between the fast light and slow light regions^30^,^32^. Reference structures comprise identical wide and narrow strip waveguides (in the monomodal section, the size of the waveguide is 260 nm × 450 nm), so the normalization of the transmission takes into account the input and output coupling from the chip. The quality of the fabrication can be assessed from the SEM images and by the PhCW transmission in dB units. At short wavelengths, the decrease of the transmission is related to the crossing of the light-line, region where out-of-plane scattering becomes important. At longer wavelengths, light group velocity decreases and backscattering reduces the transmission. Strong variations of the transmission are typical of multiple scattering, as well known in PhCW^11^. Especially, the peak around 1590–1595 nm is the signature of strong coherent back- and forward-scattering that happens close to the band edge. This behavior have been investigated previously and interpreted as caused by disorder^12^. As the waveguides are long, this kind of localized effects are more likely to occur. Considering the losses of the four couplers, slot PhC losses of a few tens of dB/cm in the fast light (λ ≈ 1560 nm, n_g_ ≈ 4) are coarsely estimated from Fig. 1. The dispersion engineered structures have been simulated by the plane wave expansion method using the MPB software^33^ for a quasi-TE polarization. Eigenmodes have been calculated in a unit cell reproducing the geometrical parameters described in the fabrication. A mesh resolution *a*/20 = 21 nm and subpixel smoothing were used.

**Figure 2:**
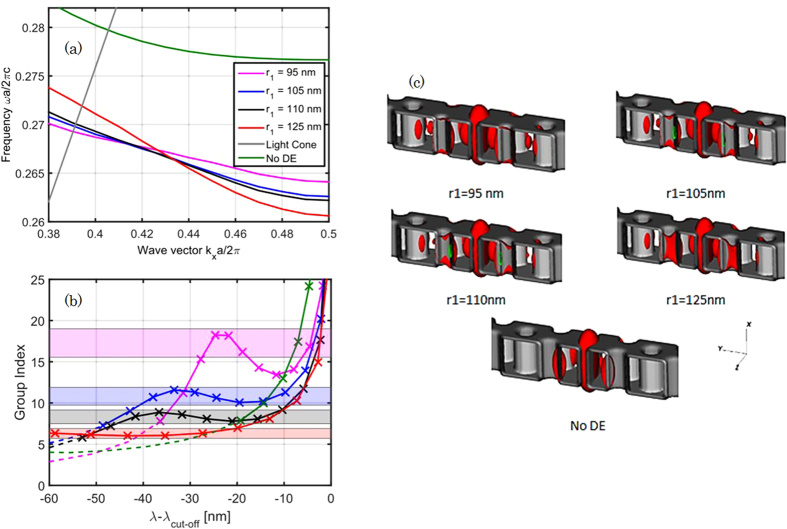
Frequency band of the dispersion engineered (DE) mode for different waveguides with their respective guided |*E*|^2^ mode profile at k = 0.46 * 2π/a. (**a**) Shows the different bands by sweeping *r*_1_ compared to a non-engineered structure. In (**b**), *λ*_*cut*_ designates the wavelength where the forbidden gap starts, dashed lines are related to the leakage above the light line, and color blocks represent the <ng> ±10% ranges. In (**c**), the different modes are displayed.

The related results are shown in (Fig. 2a). From the depicted dispersion curves, there is a clear increase of the *ω*(*k*) slope when the first row hole radius increases, which means that a lower group index is achieved. (Figure 2b) shows the group index as a function of the wavelength for each geometry. The dashed lines represent the leaky wavelengths above the light line and the color boxes have been used to calculate the figures of merit of the structures that are summarized in Table 1. From the mode distribution which remains very well confined in the slot for all the cases and the moderately high targeted group index values around 15–20, the optical losses and thus the experimental transmission levels are expected to be very similar. Note that the mode is for a large part confined within the slot, therefore these relatively modest group indices would actually correspond to slow light enhancement of *S* = 10–14 in the cladding. This is why it is important to have a large part of the field in the cladding and a material with interesting third order nonlinear properties at telecom wavelengths.

### OCT measurements

An interferometric optical coherent tomography (OCT) technique was implemented, allowing to measure the time-frequency reflectance using a partially coherent source that allowed to extract quantities such as the complex frequency-dependence reflectance or the group velocity from fringes analysis^23^,^27^,^34^,^35^.

**Figure 3:**
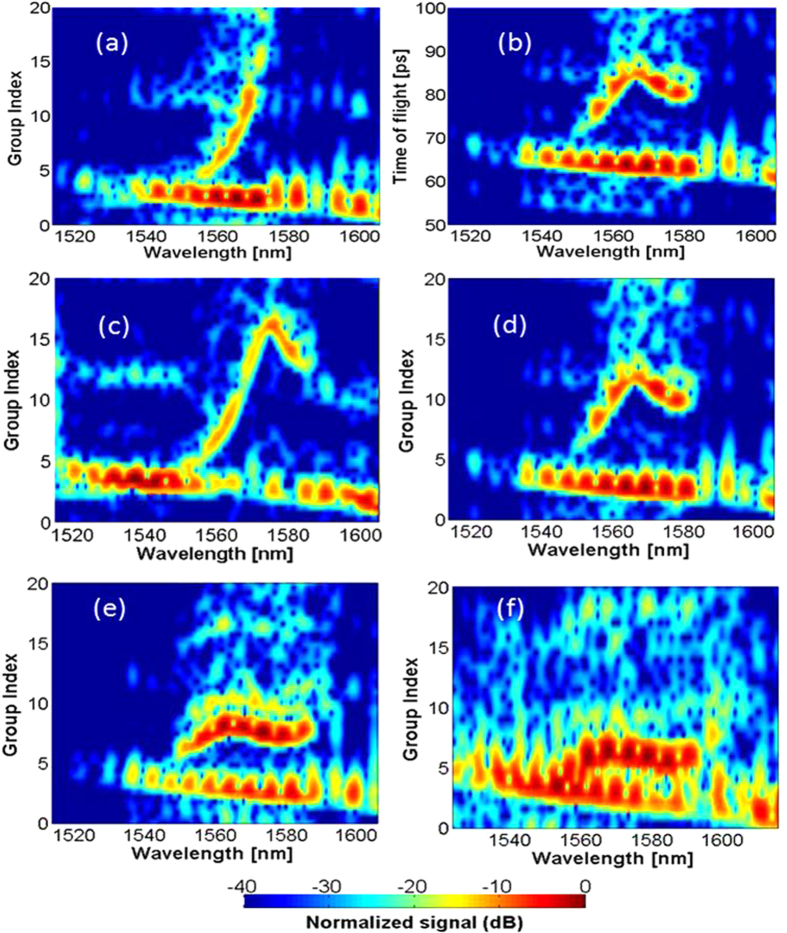
Spectrograms of different SPhCW: (**a**) Non-engineered, (**b**) Delay map directly measured corresponding to (**d**) after applying the relation (1). (**c–f**) the dispersion diagrams by sweeping the first hole radius (**c**) *r*_1_ = 95 nm. (**d**) *r*_1_ = 105 nm. (**e**) *r*_1_ = 110 nm. (**f**) *r*_1_ = 125 nm.

The spectrograms of 700 μm-long SPhCW are shown in Fig. 3. The delay was calibrated using a reference sample without SPhCW. The contributions from the access waveguides were taken into account in the final measurement. So, to extract only the photonic crystal information, the group index of the access was conservatively assumed equal to 4 (in accordance with numerical simulations). The group index of the device under test was then obtained by the deduction of the delay introduced by the access as


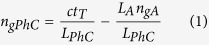


where the sub-indices PhC and A stands for Photonic Crystal and accesses, respectively, and *t*_*T*_ is the total delay measured with the spectrogram.

The transmission level for long structures demonstrates the good fabrication quality; and several of the investigated structures clearly exhibit a plateau group index, demonstrating the potential of such waveguides for applications requiring low GVD. Note that two lines can be seen on the spectrogram: the one at lower group index that decreases continuously corresponds to the TM mode intentionally coupled in. The dispersion of this mode is very close to the expected behaviour of an index-guided waveguide^27^, meaning that it is not affected –i.e. not guided- by the PhC design, and thus it serves as an internal reference to determine more precisely the actual group index. No specific post-processing regarding the polarization of light was thus performed. Regarding the thickness of the dispersion curve due to experimental uncertainties, an uncertainty criterion corresponding to a decay of 10 dB in the transmission for a given wavelength was chosen, corresponding to an estimation of *n*_*G*_ with an average accuracy of ±0.6. Let note that Fig. 3 gives also interesting qualitative features about the propagation losses. Indeed dispersion engineering does not only modify the dispersion properties of slow light modes, but also impacts their propagation losses^36^,^37^.

## Discussion

**Figure 4:**
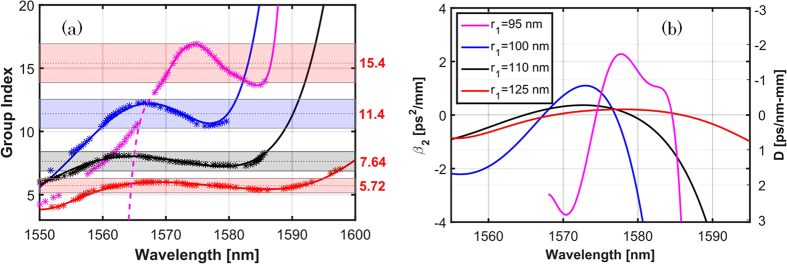
(**a**) Extracted Group Index for the different spectrograms withing 10 dB (marked with bars) and a 7^th^ order polynomial fit. (**b**) Dispersion parameter (**a**) and second order dispersion (**b**) as a function of the wavelength extracted by the analytical derivative of the fitted polynomials shown in (**a**).

Using these direct time of flight measurements, directly related with the group index, a seventh order polynomial fit was done following the higher transmission points for the TE mode in order to retrieve the group index as a function of light wavelength. As visible from the comparison between Figs 2(b) and 4, the close agreement with the simulation is remarkable. From simulations, the effective area in the slot region^23^ is estimated to be around 0.03 μm^2^. The fraction of dielectric energy confined within the slot^38^ is around 27% at *k* = 0.46 * 2π/*a* for all the structures, and about 52% of the energy is contained in the cladding region (including the holes and the field above silicon). Small effective area, hence good confinment, shows the potential of the investigated waveguide platform, where the light-matter interactions with the slot material can be enhanced.

The group velocity dispersion *β*_2_ coefficient^39^ was then estimated through first derivative of the fit:


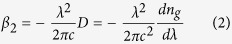


where D is the standard optical fibre dispersion parameter, yet usually reported in ps/(nm-mm) for integrated waveguides. Note that under normal dispersion (*β*_2_ > 0), optical pulse broadens along light propagation. However, in the anomalous case (*β*_2_ < 0), an optical soliton can form and the pulse hence propagates without further distortions^39^. To properly exploit the benefits of slow light, the dispersive compensation condition needs to be achieved over a large spectral bandwidth. For instance, engineered chalcogenide planar waveguides have shown supercontinuum generation^40^.

From Fig. 4, it is seen that the structures exhibit very interesting dispersion features, especially in the large group index regions. Both normal (positive) and anomalous (negative) GVD can be obtained and some SPhCWs exhibit a almost flat *n*_*g*_ plateau over more than 20 nm bandwith. This is encouraging because state of the art nonlinear effects in integrated optics have been demonstrated in the anomalous dispersion regime and for relatively modest NDBP products^15^,^41^,^42^,^43^, demonstrating the important role played by the optical losses (both linear and nonlinear) that must be meticulously treated. As the proposed structures offer wavelength windows in the anomalous regime with moderate losses, they are convenient for nonlinear applications. These results show that the advantages of slow light structures, but optimized for low propagation losses and with careful dispersion engineering, can thus be obtained in such hybrid structures filled by various materials. This fact opens broad perspectives in view of the richness of materials that can be drop-casted, spin-coat, or grown on top of silicon wafers including nonlinear and active materials. PhC waveguides are highly dispersive, consequently the geometry controls the behavior of the light propagating into the structure an takes over by several orders of magnitude the natural material dispersion. Consequently, the material dispersion can be neglected if the material is not operated at a wavelength close to a direct transition. In the present work we focus on potential claddings with a base refractive index of about 1.5. However, the finding associated with the design we present here remain valid for filling material whose index is lower than 1.8, above, the contrast between the slotted PhC and its surrounding is too weak to allow propagation and proper confinement of light. The exact impact of variations of the slot index around 1.5 and how it could be pre-compensated by the design, will be detailed elsewhere.

The sensitivity of the dispersion curves is remarkable considering that the hole radius changes only by 5 nm in the first row and that the central wavelength of the flat-band remains almost unchanged and around 1570 nm. The dispersion parameter of the waveguides exhibits interesting anomalous and normal zones, including two zero group velocity dispersion points. As the radius increases, the dispersion becomes flatter and tends to zero over larger intervals. The flat behavior is explained by the change of *n*_*G*_ over short wavelength ranges, that is smoother for larger radii. On the other hand, the zero dispersion points are obtained in maxima and minima of the *n*_*G*_ curve, and the maxima and mimina of *D* corresponds to a concavity change of the group index (zero group velocity dispersion point). We have made a quantitative exploitation of the results highlighted in Fig. 4 in order to evaluate the SPhCW main factors of merit. Results are summarized in Table 2, showing a decreasing bandwidth (Δ*λ*) as a function of the increasing of the average group index. Estimated NDBP values range between 0.133 and 0.156, i.e. extremely large for such slotted waveguides limited by a silica and top cladding light lines. By comparing with the theoretical values shown in Table 1, we see that the fabrication errors (exact hole positions, sidewall roughness, etc.) tend to decrease the actual PhC performances: designs with the best features (in theory) have often (in practice) very low fabrication tolerance. Therefore it is also important to assess the robustness of the PhC design –namely the sensitivity of the PhC’s features to small variations of the design parameters-, and not to rely only on its theoretical performances. The designs with *r*_1_ = 95 nm and *r*_1_ = 105 nm appear to be robust to fabrication errors.

In order to estimate the field enhancement in the photonic crystal, assuming a structure with *n*_*G*_ = 15, the third order nonlinear properties of silicon is increased by a factor 
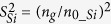
 of around 18.6. In the same time, a material filling the slot with the same refractive index used in these experiments encounters a 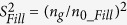 ≈ 107.0 enhancement. So, even with modest group indices corresponding to acceptable losses through long propagation lengths for practial applications (700 μm, here), the enhancement experienced by the slot material in ultra-small effective areas is very promising regarding highly nonlinear liquids and polymers with low refractive indices at *λ* = 1550 nm exhibiting ultra-fast response^44^,^45^,^46^. For instance, in the lower ng obtained experimentally (*n*_*g*_ ≈ 5.72), representing the best waveguide in terms of transmission, the expected slowdown enhancement would be of 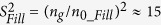. The resulting total effective nonlinearity experienced by the light is then a result of the slow light enhancement S^2^ combined with the light confinement (i.e. the effective area). Sometimes, large slow light enhancements are mitigated by larger effective area; this is not the case here. To give out some numbers, if we consider the polymer PSTF66 (n_2I_ = 2.8 * 10^−18^ m^2^/W)^25^ which has modest Kerr nonlinearity, then the waveguides presented in this article, particularly for the case *r*_1_ = 105 nm, would exhibit a Kerr nonlinearity up to 1259/W/m: 165/W/m comes from the cladding itself while the surrounding silicon PhC structure participates to 1094/W/m. But if other material with better nonlinear properties, but still at the same refractive index, such as nanocomposites (n_2I_ ≈ 1 * 10^−16^ m^2^/W)^26^, the Kerr nonlinearity would then be 6992/W/m with (as expected) exactly the same contribution from silicon (1259/W/m) and 5898/W/m from the cladding. This highlights the importance of the cladding material.

To conclude, we demonstrate slow light operation with NDBP as high as 0.156 in silicon slotted photonic crystal waveguides with a transmission level only 5 dB lower than the one of reference SOI strip waveguides, including the in/out coupling stages that are used to excite the slow light slot optical mode. The investigated structures consist in 700 μm-long hollow core photonic crystal slotted waveguides fabricated on a non-membrane SOI wafer and filled by top cladding materials. The flexibility of the control of the slot photonic crystal waveguides dispersion properties has been experimentally demonstrated by tuning a single geometrical parameter leading to both normal and anomalous dispersion regions and the presence of zero GVD points. Flat band properties in the sense of the classical NDBP definition are obtained over bandwidth typically ranging between 20 nm and 40 nm. Considering the specific property of these slotted structures to still confine a large fraction of the light into low index materials, the present demonstration opens broad perspectives regarding the integration of SPhCWs for on-chip all-optical signal processing that benefits from enhanced third-order nonlinear effects in the anomalous and nearly-zero GVD regimes, specifically few-mW optical power four-wave mixing and solitonic effects.

## Methods

### Fabrication

The samples consist of SOI wafers (Soitec) with a 260 nm-thick silicon film over 2 μm of buried SiO_2_. Patterns have been masked by ZEP-520A resist (Zeon Chemical Co.) and written by 80 kV e-beam lithography Nanobeam NB-4 system. The writing field is set to 50 × 50 μm^2^ for a beam current of 0.5 nA and a beam step size of 2 nm. The samples are transferred by Inductively Coupled Plasma reactive ion etching process using SF_6_ and C_4_F_8_ gases, and finally mechanically cleaved. The surface of the samples is covered by a refractive liquid (Cargille) with an index of 1.45 at 1550 nm properly filling both the holes and the slot as demonstrated in previous sensing works^47^. The total length of the device is 5 mm, which includes 3 μm-wide access waveguides intended for end-fire coupling. These waveguides are tapered down along 300 μm to match the 400 nm-wide wire waveguides, which are then connected to the SPhCW through a three-stage converter.

### Transmission spectrum measurement

The optical set-up used to characterize the samples consists of a tunable external cavity laser filtered by a polarizer in order to excite only the TE mode. A monomode tapered lensed fibre is used to inject the light into the chip. At the output, the transmission is collected by a microscope objective and after sent to an all-band optical component tester CT400 obtaining the transfer function.

### Measurement of the optical delay

The OCT based setup used to measure the photonic delay is based on a tunable laser source (TUNICS, Yenista Plus S) and on a Mach-Zehnder interferometer where one of the propagating arms includes the device under test and the other one a known propagation path. By the means of a Fourier treatment, the measured interferogram could be digitally processed and the photon delay of one arm with respect to the other could be estimated by the use of phase and amplitude information. The time axis is calibrated with a reference spectrogram from a reference strip waveguide^23^,^27^. The temporal window resolution is set at 1 ps, which corresponds for a 5 mm-long sample to a group index resolution of 0.062.

### Computation of effective Kerr nonlinearity

Both the cladding and the surrounding silicon PhC structure participate to the effective nonlinearity of the waveguide. Knowing the field distribution 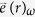 of the Bloch mode at the angular frequency ω, one can compute the total effective Kerr nonlinearity, expressed as:


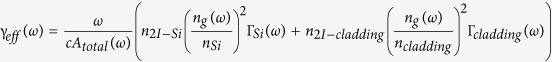


where n_2I−x_ is the nominal material Kerr nonlinearity for either the filling material or the silicon. The total effective area A_total_ is defined as A_total_^−1^ = A_Clad_^−1^ + A_PhC_^−1^. The cladding (resp. PhC) area are defined as 
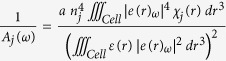
 where n_j_ is the cladding (resp. PhC) refractive index and χ(r) is a function that is valued 1 in the cladding (resp. PhC) and 0 everywhere else. Integrals are meant over a single PhC lattice, whose lattice parameter is “*a*”. According to the definitions given above, the confinement factor Γ_cladding/Si_ is then defined as Γ_j_ = A_total_/A_j_. We have by construction 0 < Γ_cladding/Si_ < 1. Further information regarding how to define effective nonlinearity (and thus the consecutive effective parameters used in the nonlinear propagating equations) for PhCs can be found in ref. 48, 49, 50.

## Additional Information

**How to cite this article**: Serna, S. *et al*. Experimental GVD engineering in slow light slot photonic crystal waveguides. *Sci. Rep*. **6**, 26956; doi: 10.1038/srep26956 (2016).

## Figures and Tables

**Table 1:** Parameters calculated from simulations.

*r*_1_[nm]	<*n*_*G*_> (±10%)	Δ*λ*[nm]	NDBP
95	17.3 (±1.7)	9.8	0.108
105	10.8 (±1.1)	32.1	0.221
110	8.32 (±0.83)	35.1	0.186
125	6.30 (±0.63)	49.9	0.200

**Table 2:** Parameters extracted from the measurements.

*r*_1_[nm]	<*n*_*G*_> (±10%)	Δ*λ*[nm]	NDBP
95	15.4 (±1.5)	13.9	0.136
105	11.4 (±1.1)	21.5	0.156
110	7.64 (±0.76)	31.4	0.153
125	5.72 (±0.57)	36.6	0.133
